# A New Local Fractional Entropy-Based Model for Kidney MRI Image Enhancement

**DOI:** 10.3390/e20050344

**Published:** 2018-05-05

**Authors:** Ala’a R. Al-Shamasneh, Hamid A. Jalab, Shivakumara Palaiahnakote, Unaizah Hanum Obaidellah, Rabha W. Ibrahim, Moumen T. El-Melegy

**Affiliations:** 1 Faculty of Computer Science and Information Technology, University Malaya, Kuala Lumpur 50603, Malaysia; 2 Electrical Engineering Department, Assiut University, Assiut 71515, Egypt

**Keywords:** local fractional, entropy, MRI, image enhancement

## Abstract

Kidney image enhancement is challenging due to the unpredictable quality of MRI images, as well as the nature of kidney diseases. The focus of this work is on kidney images enhancement by proposing a new Local Fractional Entropy (LFE)-based model. The proposed model estimates the probability of pixels that represent edges based on the entropy of the neighboring pixels, which results in local fractional entropy. When there is a small change in the intensity values (indicating the presence of edge in the image), the local fractional entropy gives fine image details. Similarly, when no change in intensity values is present (indicating smooth texture), the LFE does not provide fine details, based on the fact that there is no edge information. Tests were conducted on a large dataset of different, poor-quality kidney images to show that the proposed model is useful and effective. A comparative study with the classical methods, coupled with the latest enhancement methods, shows that the proposed model outperforms the existing methods.

## 1. Introduction

As one’s living style changes, various unexpected health issues arise in the same proportion. To find solutions to such diseases, new devices and systems have been developed. Despite the availability of new systems that help find cures to diseases, new problems have appeared due to complex diseases and the inherent limitations of the systems’ capacities [1]. One such sensitive issue is kidney segmentation and its shape analysis for disease identification, in which MRI systems generate very poor quality images in the initial phase of body scan procedure. Although the systems generate good quality images after some time, it is hard to predict an accurate time and a suitable number of images to acquire good-quality MRI images. As a result, finding quality images is time-consuming and labor-intensive. Besides, common diseases such as Acute Kidney Injury (AKI) and Chronic Kidney Disease (CKD) affect the quality of the images and the shape of the kidney identified [1]. This is due to the swelling of neighboring tissues of the kidney. As a result, this makes the process of kidney enhancement more complex and challenging. 

**Figure 1:**
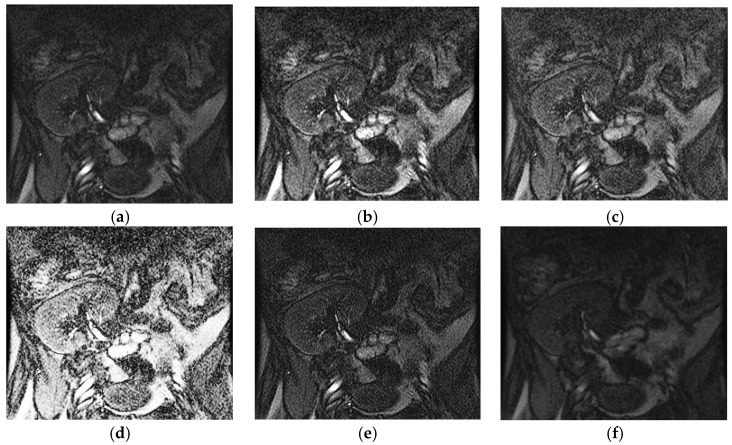
Challenges for kidney image enhancement. (**a**) Input low contrast kidney image (**b**) AIV [2], (**c**) CLAHE [2], (**d**) HISTEQ [2], (**e**) Riesz fractional [2] and (**f**) Tsallis entropy [4].

The image shown in Figure 1a is an example of a poor quality image generated by an MRI system in which the pixels of the kidney’s area and other surrounding tissues appear the same. Figure 1b–f, respectively, shows image enhancement results from various methods including the Adjust Intensity Values (AIV) [2], the Contrast Limited Adaptive Histogram Equalization (CLAHE) [2], the Histogram Equalization (HISTEQ) [2], the Riesz fractional [3], and Tsallis entropy method [4]. It is noted that methods such as the AIV, CLAHE Riesz fractional, and Tsallis entropy did not improve the edge details of the kidney compared to the input image (Figure 1a); on the other hand, the HISTEQ method enhanced the details of both kidney’s boundaries and other surrounding regions in the image. AIV, CLAHE, and HISTEQ are classical methods that are often taken as the basis for the development of newer methods [2]. However, these methods are best employed when the whole image is to be enhanced, thus affecting the image globally. Although the Riesz fractional and Tsallis entropy fractional methods are effective for enhancing low-contrast text images, they are not made for kidney images enhancement [3]. As a result, we can confirm that the existing state-of-the-art methods achieve good results for enhancing images, albeit with a global effect on the image. However, in the case of kidney imaging, the images contain different levels of quality at different regions; therefore, there is a need for developing a model that considers local information for enhancing edge details in kidney MRI images.

Existing studies have proposed a number of methods for kidney image enhancement. For example, Kang et al. [5] proposed a new feature-reduction method for ultrasound B-mode imaging using the multiscale analysis. The boundaries and borders are emphasized via edge coherence and contrast enhancement. The whole process involves diffusion filtering for reducing the effect of speckle noise. However, the main goal of this method is to reduce speckle noise rather than enhancing low contrast images or poor quality images as proposed in this work. Zhang et al. [6] proposed an efficient, small blob-detection method using intensity, local convexity, and shape information. To identify and detect the blob, the method enhances the low contrast information in the kidney images. The method explores local convexity for enhancing details in the kidney images. The scope of the method is limited to a specific dataset and its applications. In a different study, Baselice et al. [7] proposed an enhanced Wiener filter for ultrasound image restoration. The main target of the method is to reduce the speckle noise effect by exploring Local Gaussian Marko Random filed. In addition, the method adapts the Wiener filter, such that it tunes its kernel to combine the edges and for preservation with effective noise reduction. However, the method is developed for a specific application, which is noise removal, not enhancing poor quality kidney images. Koyuncu and Ceylan [8] proposed a hybrid tool for the enhancement of abdominal CT images as a pre-processing step before tumor segmentation. The method proposed block matching and 3D filtering for the denoising and elimination of Gaussian noise. Then, linking the spiking cortical model has been used for the removal of internal fat tissue. Finally, the method uses the Otsu algorithm for removing redundant parts of the image. The method focuses on particular noise and parts of the image for enhancement. Gungor and Karagoz [9] proposed the homogeneity map method for speckle reduction in diagnostic ultrasound images. The local homogeneity map is generated based on local statistics of the window formed. The method explores diffusion and gradient information, along with statistics for enhancing the images. However, the method works as denoising filter for removing noise. Recently, Roy et al. [10] proposed a model for text detection and recognition in video frames using Fractional Poisson enhancement. The method explores generalized fractional calculus to enhance the quality of the images that are obtained by Laplacian operation. The method considers the edges and their neighboring information in deriving a mathematical model. Again, the method is developed for removing the effect of Laplacian noise but not for improving poor quality kidney images. Similarly, Raghunandan et al. [3] proposed the Riesz fractional-based model for enhancing license plate detection and recognition, which usually suffers from low contrast and low resolution. The model implements the convolution operation between the Riesz fractional derivative and the input images by enhancing the edge strengths in it. The model is good for the images with text information but not for the images of kidney, which suffer from poor quality affected by unknown causes. 

In the light of the above discussion, it is noted that most methods focus on denosing and speckle noise removal for enhancing the kidney images. These methods explored different filters for reducing noise. Besides, the methods use binarization algorithm for obtaining a binary image, such that the method can enhance high contrast information in the kidney images. These methods are good for enhancing the whole image but not for the enhancement of local information in the image. Similarly, few methods addressed the issues of low contrast and poor quality of images by proposing fractional-based models. However, the scope of such methods is limited to specific applications such as text detection and recognition. Therefore, we can conclude that none of these methods have addressed the issue relating to the poor quality of kidney images and explored generalized models for enhancing such images. In addition, most methods use global information for enhancing the images but not local information. Hence, there is a scope for developing a generalized model for enhancing poor quality kidney images affected by several adverse factors such as MRI systems, diseases, and noise. Thus, in this paper, we propose a generalized and new model for enhancing poor quality kidney images based on Local Fractional Entropy (LFE). Motivated by the methods [3,10,11,12,13] that indicate that the Fractional calculus has an ability to enhance low contrast information, we explore the same methods in new way for addressing the issue of poor quality kidney images. The main advantage of LEF is that if the image contains small change in the intensity values, it is capable of detecting them as edges through probability and local entropy. More details are discussed in the subsequent section. 

The remainder of this paper is prepared as follows: Section 2 describes Local Fractional Entropy for kidney images, Section 3 discusses the experimental results for validating the proposed model, and, finally, Section 4 presents the conclusion and future work. 

## 2. Proposed Model 

As pointed out in the previous section, when there is a significant difference between intensity values in the image, it is easier to enhance the edge details. However, when there is a small change in intensity values due to factors such as noise, disease, neighboring tissues, and scanning systems, enhancing edge details can be challenging. Inspired by methods [3,10,11] in which fractional calculus has been explored for enhancing text detection and recognition performance, we propose the local fractional entropy model for extracting the above observation. 

### Local Fractional Entropy 

For each pixel in the image, the proposed model derives local fractional entropy based on frequency details of the input image. As a result, the proposed model enhances each pixel in which the gray-level changes are insignificant without affecting high frequency details.

For a continuous function *φ* in [*a*, *b*], and for a variable *u* in [*a*, *b*], the local fractional integral is defined by the following formula [14,15,16,17].

(1) $$I^{\left(\alpha \right)\;}\varphi \left(u\right)=\frac{1}{\Gamma \left(1+\alpha \right)}\;\int_{a}^{b}\varphi \left(u\right)\;{\left(du\right)}^{\alpha },$$

in which Γ is Euler gamma function, and 0 < *α* ≤ 1 is the fractional power operator. 

The discrete form of (1) is given by:

(2) $$I^{\left(\alpha \right)\;}\varphi \left(u\right)=\frac{1}{\Gamma \left(1+\alpha \right)}\;\underset{\Delta u_{k\to 0}}{\lim}\sum_{k=0}^{n-1}\varphi \left(u_{k}\right){\left(\Delta u_{k}\right)}^{\alpha },$$

in which $\;\Delta u_{k}=u_{k+1}-u_{k},u_{0}=a.$

Recently, fractional entropies have been suggested by many authors (see [18,19]) for solving fractional nonlinear problems (see [20,21,22,23]). 

We consider Tsallis entropy as local fractional entropy for enhancing the fractional integral operators. The pixel’s probability in the input image is denoted by *φ*. The Tsallis entropy is defined as

(3) $${Ε}_{\alpha }\left(\varphi \left(u\right)\right)=\;\frac{\int_{a}^{b}{\left(\varphi \left(u\right)\right)}^{\alpha }\;du\;-1\;}{1-\alpha }.$$

Hence, in the discrete form we have

(4) $${\;Ε}_{\alpha }\left(\varphi \left(u\right)\right)=\;\frac{1}{1-\alpha }\;\left(\overset{n-1}{\underset{k=0}{\sum }}\;\varphi^{\alpha }\left(u_{k}\right)-1\right)$$

By considering the derivative with respect to *φ* for both sides of (4), we obtain

(5) $$\;{\overset{´}{E}}_{\alpha }\left(\varphi \left(u\right)\right)=\;\frac{\alpha }{1-\alpha }\;\overset{n-1}{\underset{k=0}{\sum }}\;\varphi^{\alpha -1}(u_{k})\;.$$

The power function $\varphi^{\alpha }$ in (2) has the following local fractional integral:

(6) $$\;I^{\left(\alpha \right)\;}\varphi^{\alpha }\left(u\right)=\frac{1}{\Gamma \left(1+\alpha \right)}\;\underset{\Delta u_{k\to 0}}{\lim}\overset{n-1}{\underset{k=0}{\sum }}\varphi^{\alpha }\left(u_{k}\right){\left(\Delta u_{k}\right)}^{\alpha }\;.\;$$

In our study, we consider distance between pixels is equal to 1, so that the approximation of limit part of (6) will be as follows:

$$\underset{\Delta u_{k\to 0}}{\lim}{\left(\Delta u_{k}\right)}^{\alpha }=1.$$

Thus, we have

(7) $$\;I^{\left(\alpha \right)\;}\varphi^{\alpha }\left(u\right)=\frac{1}{\Gamma \left(1+\alpha \right)}\;\underset{\Delta u_{k\to 0}}{\lim}\overset{n-1}{\underset{k=0}{\sum }}\varphi^{\alpha }\left(u_{k}\right)\;.\;$$

By taking the derivative with respect to *φ* for both sides of (7), we attain

(8) $${\overset{´}{I}}^{\left(\alpha \right)\;}\varphi^{\alpha }\left(u\right)=\frac{\alpha }{\Gamma \left(1+\alpha \right)}\;\underset{\Delta u_{k\to 0}}{\lim}\overset{n-1}{\underset{k=0}{\sum }}\varphi^{\alpha -1}\left(u_{k}\right)\;.$$

To consider the local fractional entropy for image enhancement as the convolution of (5) and (8), we need the following preparation:

(9) $$G:={\overset{´}{I}}^{\left(\alpha \right)}\varphi^{\alpha }\left(u_{k}\right)*{\overset{´}{Ε}}_{\alpha }\left(\varphi \left(u_{k}\right)\right).$$

Thus, we obtain the local fractional convolution operator

(10) $$G=\;\frac{\alpha^{2}}{\left(1-\alpha \right)\Gamma \left(1+\alpha \right)}\left(\overset{n-1}{\underset{k=0}{\sum }}\frac{1}{\varphi^{1-\alpha }\left(u_{k}\right)}\;\right),\varphi \left(u_{k}\right)\neq 0.$$

From (10), we have the following enhancing coefficient of local fractional entropy of order α (this is the contribution of our study):

(10) $$G_{k}=\;\frac{\alpha^{2}}{\left(1-\alpha \right)\Gamma \left(1+\alpha \right)}\varphi_{k}{}^{\alpha -1}\;,\;k=0,1,2,\ldots ,n-1,$$

in which $\varphi_{k}{}^{\alpha -1}=\varphi {\;}^{\alpha -1}\left(u_{k}\right)$ is the local fractional probability of the pixel. 

By using the local fractional entropy operator ($G_{k}$), we construct a Local Fractional Entropy (LFE). The enhanced image IF is given by:

(11) $$\;I_{F}=G_{k}\cdot I$$

in which *I* is the input image. 

The fractional power values (α) of the proposed G operator is defined by the range of 0 < α ≤ 1.

**Figure 2:**
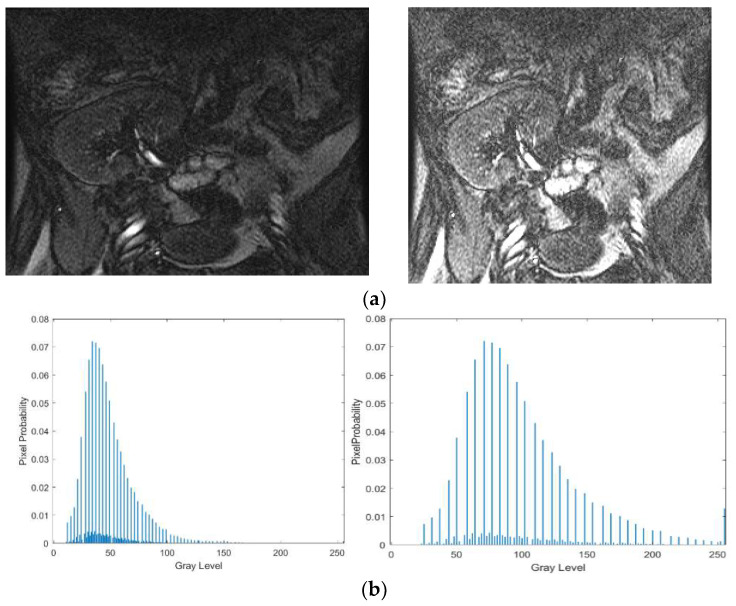
Contrast increases after enhancement by the proposed method. (**a**) Input Kidney image and its enhancement image by the proposed method; (**b**) Histogram before enhancement (**Left**); Histogram after enhancement (**Right**).

**Figure 3:**
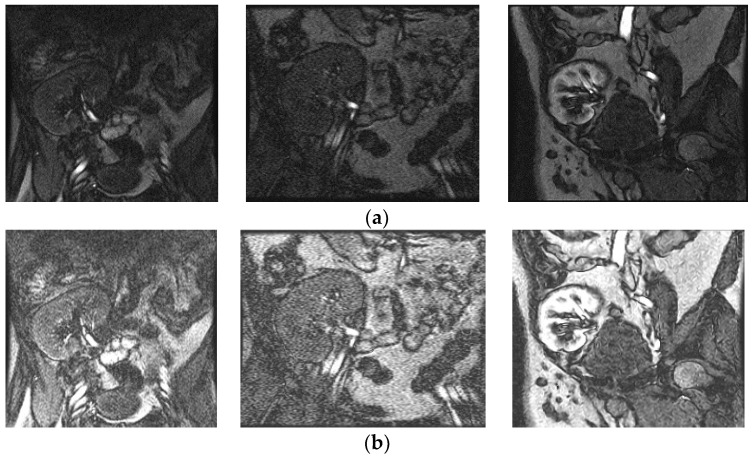
The result of proposed enhancement model. (**a**) Input poor quality kidney images and (**b**) enhanced images.

The above steps work well, because contrast enhancement of the input image is determined at each pixel depending on the probability of the pixel, which controls the changes in the gray values of the input image. Figure 2 shows a poor-quality input image with its enhanced counterpart, as well as graphs of their distribution of probability of pixels. It is noted from the enhanced image shown in Figure 2a that the contrast between background and boundary pixels of the kidney image is increased compared to the input image. This shows that the proposed model improves the overall quality of the image. It is evident from Figure 2b that the distribution of pixels’ probabilities for the input image before enhancement appears to be dense. Similarly, the distribution of pixels’ probabilities appears to be scattered in the enhanced image, which means the contrast has been stretched. Therefore, we can conclude that the low-contrast pixels, which represent boundaries of the kidney, are enhanced and hence result in scattered probability distribution with the same frequencies of the input image. Figure 3 shows the enhancing effect of the proposed model for a few more poor-quality kidney images. Input images are shown in Figure 3a, while the enhanced images are shown in Figure 3b. 

## 3. Experimental Results

To the best of our knowledge, there is no standard dataset for kidney image enhancement in the existing literature. We have assembled our own dataset from an existing dataset of kidney MRI images provided Mansoura University Hospital, Egypt [24]. Each set captures the kidney of a patient suffering from a kidney disease. As mentioned in the introduction section, the MRI system generates very poor-quality image slices at the beginning of a body scan. Thus, we manually chose the first 10 poor-quality image slices from each patient. This gave us a total of 100 images from 10 datasets of different patients. It is noted that each image poses different level of quality issues. Therefore, we believe the considered dataset is complex and ready to be used for evaluation, as it covers wide range of poor quality kidney images. 

Since the dataset is new, creating ground truth is not an easy task; therefore, we prefer to use standard, no-reference measures that do not require ground truth images, namely, BRISQUE [25] and NIQE [26]. The BRISQUE, which is a no-reference image quality measure, compares the image that is to be analyzed with a default model calculated from different images of natural scenes. This outputs non-negative scalar value for every input image; images are usually in the range of [0, 100]. Lower values represent better perceptual quality of image. In general, BRISQUE predicts the score with the help of a support vector regression model. Naturalness Image Quality Evaluator (NIQE), which is also a no-reference image quality measure, calculates score for an image using the naturalness image quality evaluator (NIQE). A smaller score specifies the best perceptual image quality.

**Figure 4:**
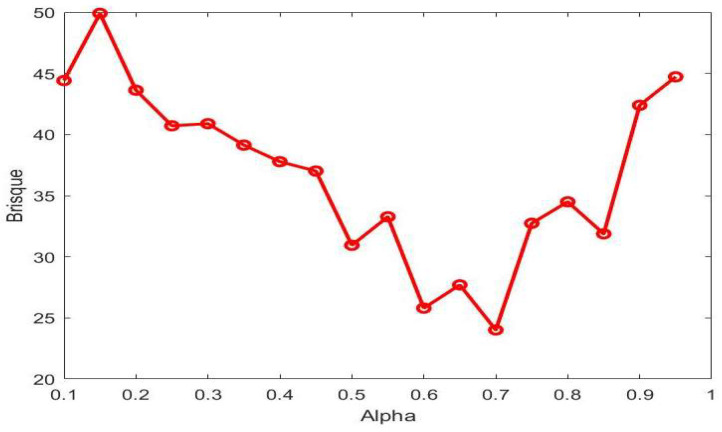
Determining the value for α empirically. Average BRISQUE measure of predefined samples is calculated for different values of α.

In the proposed model, the key parameter is α, in which the performance of the proposed model changes according to its value; therefore, we compute the average BRISQUE score for a predefined sample images from our dataset by varying the values of α. Changes in α will lead to changes in the probability of the enhanced image, which in turn lead to changes in the BRISQUE score. The proposed model chooses the value of α when the BRISQUE score touches the lowest value. As shown in Figure 4, BRISQUE gives the lowest score at 0.7 of the α. The same value is considered for all experimentation in this work. Note that the values of BRISQUE change rapidly with respect to small changes in α. This fluctuating behavior reflects the effect of fractal entropy on each pixel’s value of the enhanced image.

To show the efficacy of the proposed model, we implement the basic and recent methods for comparative study. We hypothesized that if the basic methods work well, the latest methods should work well too, because most of the recent methods directly and indirectly use the same basic idea for enhancement. Therefore, the proposed method is compared with the results from the classical methods [2], namely, Adjust Intensity Values to Specified Range (AIV), Contrast-Limited Adaptive Histogram Equalization (CLAHE), and Histogram Equalization (HISTEQ). These are quite common methods and considered as state-of-the-art methods for enhancement. In addition, Tsallis entropy method [4] proposed a new mathematical model by using the convolution of fractional Tsallis entropy for image denoising. Raghunandan et al. [3] proposed the fractional Riesz model for enhancing license plate images. This method explores fractional calculus for enhancing license plate images but not poor quality kidney images.

**Figure 5:**
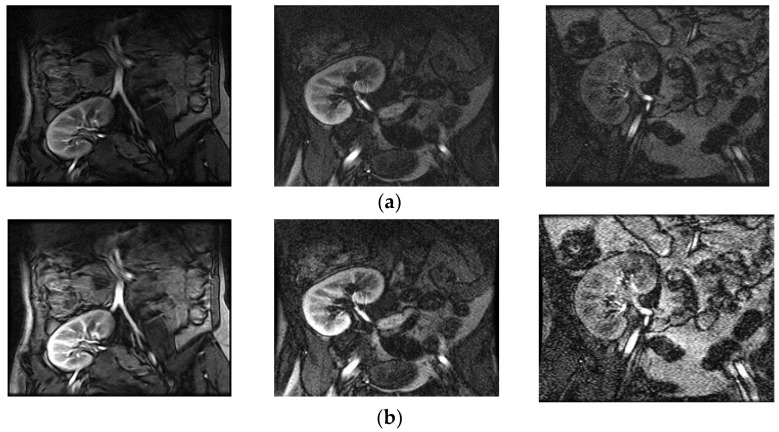

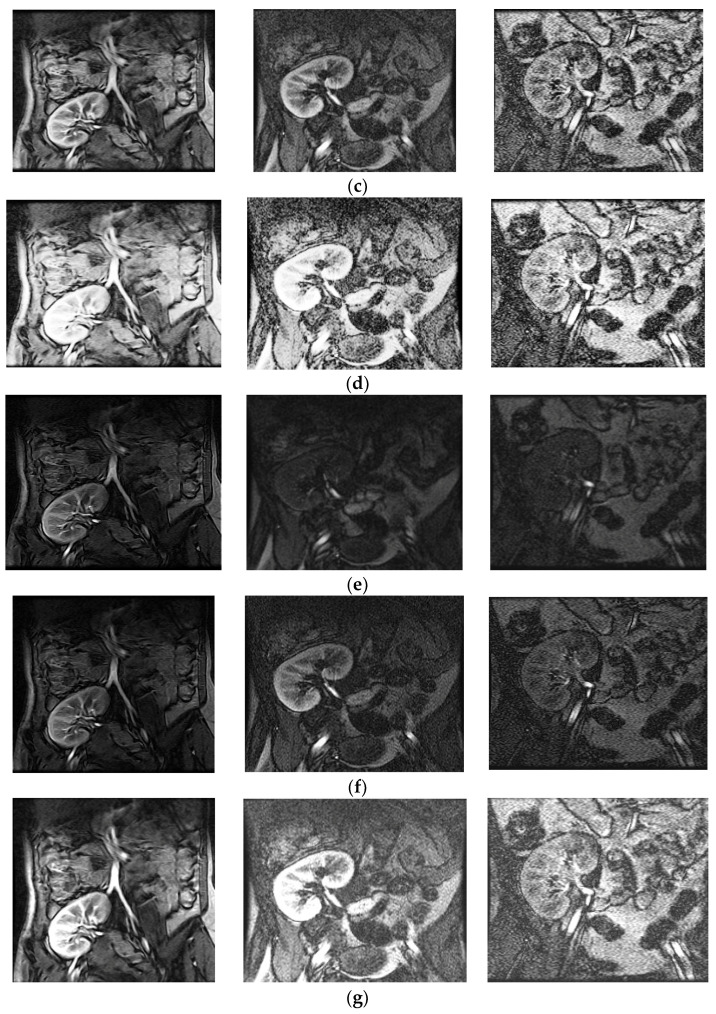
Qualitative results of the proposed and existing methods. (**a**) Input kidney images with different complexities, (**b**) Adjust Intensity Values to Specified Range (AIV), (**c**) Contrast-Limited Adaptive Histogram Equalization (CLAHE), (**d**) Histogram Equalization (HISTEQ), (**e**) Tsallis entropy, (**f**) Riesz fractional and (**g**) proposed method.

Qualitative testing results of the proposed and current existing methods for different poor quality kidney images are shown in Figure 5, in which the classical methods showed better results compared to Ragunandan et al. [3], as the latter was developed with a specific application in mind, which is enhancement of license plate images. When we compare the results of the proposed method and the basic methods, the proposed model gives better results. This is valid, because all basic methods are global methods and work well for the images affected by single cause with the same degree. On the other hand, the proposed model is suitable for images that are influenced by multiple adverse factors, resulting in varying quality at different regions of the same image. This is because the proposed model considers local information for enhancing pixels. 

Quantitative results of the proposed and existing methods are reported in Table 1, in which it can be noted that the best score of the BRISQUE and the NIQUE are obtained by the proposed model compared to the existing methods. This shows that the proposed model is better than the existing methods. In terms of BRISQUE, AIV is the second best compared to the proposed model, and CLAHE is the second best at NIQUE compared to the proposed model. In the same way, the HISTEQ and the Riesz fractional report the worst results in terms of BRISQUE and NIQUE compared to other methods. This is because the basic methods suffer from inherent limitations such as global thresholding, while in case of Riesz fractional-based method, the parameters are tuned according to the text in license plates images. Furthermore, the proposed model does not depend much on specific content of the image; rather, it explores probability of pixels using local information. Thus, this method is independent of dataset and applications. In other words, the proposed model can be used for enhancing other medical images affected by poor quality. Therefore, it can be deduced that the proposed model outperforms the existing methods in terms of applications, as well as BRISQUE and NIQUE scores. 

## 4. Conclusions

In this paper, we have proposed a new model for enhancing poor-quality kidney images based on Local Fractional Entropy. The proposed model works by considering the probability of pixels at and near the edges of the identified areas in the image. Since the proposed model uses fractional calculus, it has the ability to enhance the edge information in which there is little intensity difference rather than expecting a significant difference. In addition, the proposed model can work with images that have different regions suffering from different degrees of low quality, since it considers local information for enhancing edge pixels. Experimental results on different poor-quality kidney images show that the proposed model is effective and useful. Further, the comparative study with the state-of-the-art methods shows that the proposed model is better than existing methods in terms of BRISQUE and NIQUE. For future studies, this model could be applied to the segmentation and disease identification of kidney images.

## Figures and Tables

**Table 1:** The enhancement performance of the proposed and existing methods.

Methods	BRISQUE	NIQE
Histogram Equalization [2]	41.35	8.65
CLAHE [2]	38.85	7.08
AIV [2]	25.95	7.10
Riesz Fractional [3]	41.93	10.01
Tsallis entropy [4]	37.03	6.04
Proposed method	22.37	6.32
